# The Presence of *Legionella* in Water Used for Car Washing: Implications for Public Health

**DOI:** 10.3390/microorganisms11122992

**Published:** 2023-12-15

**Authors:** Pasqualina Laganà, Alessio Facciolà, Roberta Palermo, Osvalda De Giglio, Santi Antonino Delia, Maria Eufemia Gioffrè

**Affiliations:** 1 Regional Reference Laboratory of Clinical and Environmental Surveillance of Legionellosis, Branch of Messina, Department of Biomedical Science and Morphological and Functional Images, University of Messina, Via C. Valeria, 98125 Messina, Italy; afacciola@unime.it (A.F.); adelia@unime.it (S.A.D.); 2 Department of Health Promotion, Mother and Child Care, Internal Medicine and Medical Specialties “G. D’Alessandro”, University of Palermo, 90127 Palermo, Italy; roberta.palermo@unipa.it; 3 Regional Reference Laboratory of Clinical and Environmental Surveillance of Legionellosis, Department of Biomedical Science and Human Oncology, Section of Hygiene, University of Bari Aldo Moro, 70124 Bari, Italy; osvalda.degiglio@uniba.it; 4 Multispecialty Clinical Institute, Trauma Orthopedic Care, Via Ducezio 1, 98124 Messina, Italy; emi.gioffre@alice.it

**Keywords:** *Legionella*, car wash, occupational disease, water and aerosol monitoring

## Abstract

Although today all of the aspects of *Legionella* are better understood than in the past, in many countries the interest is still mainly focused on healthcare and tourism facilities. Other at-risk areas are less explored, such as those where workers are often in contact with water during their activities. In reality, any water system capable of producing aerosols can be considered a potential source of *Legionella* transmission, including car washes, where a large number of users work and flow through annually. From January to May 2022, 120 samples were carried out in 30 car washes located in Messina (Italy): 60 samples of water and 60 of aerosols. The aim of this investigation was to evaluate the risk of legionellosis in car washing workers exposed to potentially contaminated aerosols. To increase the probability of finding *Legionella*, the sample collections were organized on different days of the week. Of the total samples taken, 10 (8.3%) were positive for *Legionella*: seven (11.7%) water (range 100–1000 CFU) and three (5%) aerosol (range 10–150 CFU) samples. Detected serogroups were *L. pneumophila* sgr 1, 7, 10 and *Legionella gormanii*. Given the results obtained, preventative measures should be implemented in such facilities in order to protect the health of users and car wash operators.

## 1. Introduction

Legionellosis, Although today all of the aspects of legionellosis are better understood than in the past, it is still considered a concern in many countries and is related to healthcare settings and touristic-receptive structures [1,2,3,4,5,6,7,8]. Bacteria belonging to the genus *Legionella* are naturally and commonly found in freshwater environments, like lakes, rivers and streams, that represent a natural reservoir. However, these bacteria can become pathogens affecting humans because they are able to grow in many different building’s water systems like taps, air conditioners, cooling towers, hot water systems, ornamental fountains, water tanks and heaters and old and wide plumbing systems [9].

Some at-risk areas have been explored less, such as those where workers and customers are frequently in contact with water during their activities. The currently used Italian Guidelines [10], in point 6.2. “The Risk for Healthcare Workers”, consider other categories of operators such as firefighters, gardeners and horticulturists and car washers. On the basis of this consideration, each employer, in accordance with the Italian Legislative Decree 81/2008 and subsequent amendments regarding the protection of health and safety in the workplace, has the obligation to consider that the risk of legionellosis may concern both its own workers and those who attend each they are responsible for. Therefore, the obligation to carry out a risk assessment (revised at least every 3 years, subject to more stringent provisions) is reaffirmed so that all the preventative and control measures described in the previous paragraphs are implemented, not only in response to a case of *Legionella*, but before this happens, to prevent the risk. 

Despite its low notification rates in EU/EEA countries (overall 2.4 per 100,000 inhabitants), legionellosis is a relevant cause of community- and hospital-acquired pneumonia [11,12]. In the literature, an increasing number of cases of community legionellosis have been reported which are associated with the work environment [13,14,15]. 

Indeed, over the years, it has been documented that legionellosis occurs in many types of workers, specifically maintenance technicians of air-conditioning or water supply systems, dentists, workers in oil and gas installations, miners, welders, car washers, workers at biological treatment plants, land workers and workers in the forestry industry [16,17,18,19,20,21,22,23]. Nowadays, it is usually accepted that a predictable risk of exposure to *Legionellae* could be present in any situation characterized by a work activity using or storing water and where there is the possibility of creating and transmitting water droplets and aerosols that can be inhaled. Generally, the majority of occupational cases occur sporadically, and the transmission chain is very often hard to recognize. Occupational legionellosis is often considered an unpredictable accident, even though a higher risk of infection certainly exists for some categories of workers, especially those who are strictly in contact with water and aerosols, and this has created the absolute need for appropriate prevention strategies, according to the European Directive 2000/54/EC [24], which applies to all activities where workers are potentially exposed to microorganisms as a result of their work.

For this reason, legionellosis, in addition to being an infectious disease with compulsory notification, has also become an occupational health issue. Previously, in 2011, the EU-OSHA (European Agency for Safety and Health at Work) published a report titled “*Legionella* and Legionnaires’: a policy overview” [25], and a factsheet (No. 100) titled “*Legionella* and Legionnaires disease: European policies and good practices” [26], which, addressed the issue of occupational safety and health, highlighted the risk of specific categories of workers contracting certain diseases (e.g., maintenance workers air conditioning and water supply systems, workers in places with atomization machines, welders, miners, healthcare workers involved in industrial waste water treatment, car washers, etc.) and insisted that legionellosis is considered and counted among the “risks at work”, and has been included among the pathologies and the infections to monitor and deepen our understanding of and for which health surveillance is necessary.

Although nowadays the important role played by *Legionella* in workplace settings are well-known, few data are available on its incidence in these environments. Indeed, according to the origin, the typical classification of legionellosis is still divided into community-, hospital-, and travel-associated, without including work-related cases. A recent paper reviewed the incidence of legionellosis related to work activity and showed that the overall number of confirmed cases of the infection (including both Legionnaire’s Disease and Pontiac Fever) involved 805 workers, including nine deaths [27]. However, the true incidence of legionellosis, either work-related or not, is surely much higher because many cases are contracted in a sub-clinical way or remain undiagnosed. From the latter point of view, since the introduction of urinary antigen tests in the mid-1990s, underdiagnoses decreased and mortality declined, thanks to the ability to diagnose the infection early [28,29,30]. There are three main reasons for the under-diagnosis and under-reporting of legionellosis: (i) patients affected by pneumonia are suddenly treated before a microbiological diagnosis, with no need to know the real etiology of pneumonia; (ii) some diagnostic methods for legionellosis can give false negative results; (iii) in patients affected by co-morbidities and a serious clinical situation, mortality can be ascribed to their critical condition and not to the infection [31].

Based on our previous experiences, some of which have also investigated unusual sources of *Legionella* in hospital environments [32,33,34,35], we have conducted a field investigation to evaluate the risk of contracting this disease in some car washing workers and users exposed to aerosols potentially contaminated by *Legionella* in the city of Messina (Sicily, Italy).

## 2. Materials and Methods

### 2.1. Sampling

Air is not a matrix in which microbial load can be easily quantified because while some European laws establish procedures for the microbiological assessment of other environmental matrices (e.g., methods are well described for the identification of microorganisms in water samples), no specific regulations have been established for air monitoring in such environments. 

Generally, to sample biological particles in the air, both an active and a passive method can be used.

From January to May 2022, 120 samples were collected from 30 car washes located in the city of Messina (Sicily, Italy) and the nearest periphery; specifically, 60 samples of water and 60 of aerosols. Samplings were performed weekly, following an agreement with the owners. It should be noted that the water used by the car washes is from the city’s water supply system. Among the tested car washes, water recirculation was not practiced.

To evaluate the presence of *Legionella* and, consequently, to assess the risk to exposed workers and users, the first sampling was carried out on a Monday morning, after 36 h of unused pumping, before starting the activities, from the end of the rubber tube; the second sampling was carried out on a Thursday, during routine activities. 

### 2.2. Water Sampling

Samples of water were collected from taps using 5 L sterile plastic tanks according to Annex 3 of the Italian Guidelines 2015 [10] and in accordance with ISO 11731:2017 [36] “Water quality-detection and enumeration of *Legionella*. Direct membrane filtration method for water with low bacterial counts”. To increase the probability of *Legionella* isolation, each tank was kept for 3 h at 37 °C before analytical procedures. The results were expressed in colony forming units/Liter (CFU/L) of water.

### 2.3. Air Sampling

During each water sampling an active microbiological aerosol pick-up was used with the aid of the Air Ideal 3P air sampler (Biomerieux, Marcy-l’Étoile, France). This is an impactor-type device based on the principle described by Andersen [37], in which the air is drawn through a grid perforated with a series of 286 calibrated holes. The instrument is able to accommodate Petri dish plates 9 cm in diameter. The resulting air flows containing microorganisms were directed onto the agar surface of a Buffered Charcoal-Yeast Extract (BCYE) Agar plate (Oxoid Ltd., Milan, Italy).

The Air Ideal 3P was set to an air intake of 100 L/min at an impact speed lower than 20 m/s according to the impaction principle recommended by the draft standard ISO/DIS 14698-1 [38] in order to prevent the medium plates becoming overly contaminated by bacteria and moulds normally suspended in the air. The total amount of aspirated aerosol was 0.5 m^3^ (equivalent to an aspiration of 500 L of air in 5 min). The instrument was placed as near as possible to the car wash operator, to simulate true exposure. 

The number of colonies grown per plate was calculated by following the conversion table to calculate the most probable number of organisms sampled using instruments that take in air and is expressed in CFU/m^3^, as shown below (adapted from PBI international).


$$X=\frac{\mathrm{Pr}\times 1000}{V}$$

X = CFU /1000 L of air (1 m^3^).

Pr = Most Probable Number (colony count using the correction factor).

V = Volume of sampled air.

### 2.4. Isolation and Identification of Legionella *spp.*

To detect *Legionella* spp. from water samples, we used the standard procedures reported in Annex 4 of the Italian Guidelines 2015 for the prevention and control of legionellosis. Water samples were concentrated to 10 mL through the use of 0.2 µm porosity membrane filters and then incubated at 50 °C for 30 min in a thermostatic bath. Concentrated and unconcentrated samples were spread on BCYE Agar Base Medium (Oxoid) plates in duplicate, then incubated for 10 days at 36–37 °C in a moist chamber with 2.5% CO_2_. After the incubation, the suspected colonies were isolated and confirmed as *Legionella* spp. by screening their inability to grow on a culture medium deprived of cysteine. Bacterial counts were reported in CFUs/L according to the number of colonies detected per plate and to the dilutions carried out on the original sample. The isolates were further identified as *L. pneumophila* serogroups using the microagglutination kit ‘*Legionella pneumophila* antisera set 1 and 2’ and *Legionella* antisera for several *Legionella* species such as *L. dumoffii*, *L. bozemanii*, *L. micdadei*, *L. gormanii* etc. (Biogenetics, Denka Seiken Co., Ltd., Tokyo, Japan).

### 2.5. Statistical Analyses

All the obtained data were collected and analysed with Prism 4.0 software. Comparisons between groups were performed using the Chi-square test. Significance was assessed at the *p* < 0.05 level.

## 3. Results

A total of 120 samples were collected: respectively, 60 of water (30 on Monday morning and 30 on Thursday morning) and 60 of aerosol (in the same days as the sampling of water). The results are summarized in Table 1.

### 3.1. Result of Water Samples

Of the sixty analyzed water samples, seven (11.7%) were positive for the presence of legionellae: six (20%) of those collected on the Monday and one (3.3%) from the Thursday. Isolated legionellae were *L. pneumophila* sgr 1 (1 strain), *L. pneumophila* sgr 7 (1 strain), *L. pneumophila* sgr 10 (3 strains) and *Legionella gormanii* (2 strains). The limit of detection for the culturing method is 100 cfu/L.

### 3.2. Result of Aerosol Samples

With regard to samples of aerosol, of sixty collected samples, only in three (5%) was the presence of *Legionella* detected, all in samples collected on Monday. Isolated legionellae were *L. pneumophila* sgr 10 (two strains) and *Legionella gormanii* (one strain). No samples were positive among those collected on Thursday. As expected, the same legionellae isolated from water were found, in much smaller quantities, in respective aerosol samples. 

The mean concentration of legionellae isolated show significant differences between the two sampling days (*p* < 0.05).

## 4. Discussion

Clinical manifestations of legionellosis are variable in severity, ranging from a mild febrile disease to a serious and sometimes deadly pneumonia, and severity is determined by the exposure to *Legionella* species found in water. The usual form of *Legionella* transmission is the inhalation of contaminated aerosols produced in conjunction with water sprays, jets or mists [39]. Only recently has there been evidence in the literature of a possible inter-human contagion [40].

The presence of *Legionella* in aerosols and bioaerosol has been widely demonstrated by many authors [37,41,42,43,44,45,46,47], both in indoor and outdoor environments. A valid air sampling technique, together with continuous water surveillance, is able to provide many benefits in preventing legionellosis [48]. In particular, aerosols must be the object of continuous monitoring. Therefore, evaluating the air around aerosol-producing devices may provide great help in assessing the potential for *Legionella* spp. aerosolization [18], identifying possible infection sources and also assessing the distance of *Legionella* spreading [49].

Different modalities of air sampling can be performed. In the active method, a known air quantity is aspirated and conveyed on a solid or liquid collecting surface. The microbial load in the air is then measured and reported as colony-forming units per cubic meter (CFU/m^3^) [38]. At present, different instruments are available, each with some limitations, so that the choice of a device compared to another one must be based on the careful consideration of the conclusion we want to reach with the analysis and the knowledge of the limitations of the various samplers and all the possible factors capable of affecting the results [38,50]. Each active sampler provides different results when used to evaluate the air microbial load at the same time, showing a high variability. Many previously published papers aiming to compare the efficiency of different samplers showed that the final count differs from one device to the other ones [45,50]. Therefore, it can be hard to compare data collected with different samplers. The impact method on solid plate is the most commonly used active method to quantify microbial air contamination. In particular, two different impactor samplers are available, according to their different inlet characteristics: slit samplers and sieve samplers. In the first samplers, air is aspired through a single nozzle, while in the second ones, air is aspired through a plate with several holes and the particles impact on an agar medium for culture-based analysis or an adhesive-coated surface, located below the perforated plate, that can be microscopically analyzed [51]. If we are talking about an outdoor environment, we need to evaluate the direction and velocity of the wind, which, by dispersing the suspended particles, decreases the potential bacterial load that is present; other factors to consider are relative humidity, season, water temperature, etc. [52]. However, aerosol sampling techniques established to detect *Legionella*, as opposed to another microorganism, are limited by the presence of other germs and molds that, growing in this case on BCYE agar plate, often interfere with the analysis, invalidating it. It is not uncommon to find the simultaneous presence of bacteria, amoebae, molds, etc., in the water and/or aerosol samples [53,54,55].

Any water system able to produce aerosols can be considered as a potential source of *Legionella*, including car washes (where a lot of aerosols are normally produced during the work activity) which has a large number of workers and users, who are potentially affected by some chronic health conditions, causing a reduction in immune responses and then putting these subjects at a potential risk of contracting legionellosis. Appropriate measures to prevent and control Legionella growth should be implemented in such facilities to protect the health of car wash workers and also, in this particular case, of users.

Generally, in our urban areas, washing cars on the streets has long been forbidden in many communities. The reasons for this limitation were mainly due to ecological considerations, since the resulting washing water could contain oils, lubricants, tar, suspended material, heavy metals and microorganisms, which often penetrate the soil, thus posing a danger to ground waters and surface waters and representing a risk of environmental pollution. Moreover, when the cars are washed manually, much more water is used than the is used in a mechanical washing plant (ranging from 10 to 15 L to even more than 400) and this water is normally not recycled, thus increasing the waste of water. Since industrial water in a car wash facility can be microbiologically contaminated, both staff and customers must be protected from possible health hazards, especially if contaminated washing water is sprayed in closed areas without ventilation. In fact, aerosolized powder contaminated by bacteria can be inhaled, representing a potential source of infection.

Different types of car wash systems are spread across our territory: fully automated, full service (where the car is washed by special attendants) and self serve. In these three types of service, the risk differs because, while workers are exposed in the first two types, customers can be at risk in the third one. The water used at a car wash service may be used only once and drained away, or it can be collected in tanks and recycled. In this service, numerous factors can influence the growth of *Legionella* and they have to be considered. First of all, there is the high possibility of exposure to aerosols during the washing cycle (especially when warm water is used). In addition, there may be concerns about water temperature and water stagnation. Finally, there could be the potential for bacterial contamination when the water is recycled. Particularly because some compounds as soaps, dirt, oils and sediments can provide nutrients which support bacterial and protozoan growth, and it is well-known that the latter are an important vector for the survival and growth of *Legionella* [53]. Contaminated bacteria are capable of forming a biofilm inside hand-held hoses and storage tanks.

In our study, water collected from local car washes was found to be contaminated by Legionellae, even if in low concentrations. *L. pneumophila* was the most detected species. The significant difference found in samplings carried out at the beginning of the week, after the water system has not been used, compared to those conducted in the middle of the week is very interesting. This situation is in accordance with the well-known role played by water stagnation in the growth of *Legionella*, this being one of the most important critical risk factors in the spread of the disease. Another important finding was the remarkable difference found between the contamination of the water and the aerosol. Specifically, for the latter, only samples at the beginning of the week were contaminated. In terms of the detected species, *L. gormanii* must be mentioned as it was isolated from both the water and aerosols samples. *L. gormanii* is one of the 28 *Legionella* species associated with human disease and, within the species *Legionella*, is the second most commonly detected one as a cause of community-acquired pneumonia. This species was first isolated from a 64-year-old woman affected by pneumonia with some important comorbidities such as systematic lupus erythematosus and adenocarcinoma [56]. An important feature of this particular species is its clinical detection in children, while other *Legionella* species are rarely isolated in this patient group [57]. All the pneumonia cases caused by *L. gormanii* were reported in immunosuppressed patients. However, the detection of this species has also recently been demonstrated in an immunocompetent patient [58].

Our results suggest how important it is to continuously survey the safety of water used in car wash activities for both workers and users, among which we can hypothesize the presence of people affected by some health conditions (i.e., immunodepression) that put people themselves at a higher risk of developing the disease.

Despite the fact that the amount of *Legionella* that we found in the aerosol is considerably low, we can hypothesize a certain risk for both customers and users considering the remarkable exposure to aerosols produced during the activity phase of the machinery responsible for car washing. Therefore, an efficient air sampling technique, along with a continuous water surveillance, is beneficial in preventing legionellosis in this particular setting. Monitoring the air around the aerosol produced, using instruments or other tools, may support in understanding the maximum potential for *Legionella* spp. aerosolization, ascertaining possible infection sources, and evaluating the distance that *Legionella* has spread. In order to bypass the limits of air sampling, alternative methods can be used to increase the sensitivity and the possibility to detect *Legionellae* in the air. In this way, liquid sampling methods such as (Coriolis^®^μ), a portable cyclone-based air sampler for liquid medium, used to quantify *Legionella* in bioaerosols can be used [59]. This method can increase the detection of legionellae as it avoids the growth of molds that can overwhelm the development of legionellae in solid plate. Moreover, the use of molecular detection methods, such as PCR and Next Generation Sequencing (NGS), can further increase the detection of these bacteria [60].

Some countries have elaborated and issued specific guidelines and protocols for managing the risk of legionellosis having car washing systems as source of infection. In June 2008, the Water Management Society issued some recommendations (revised in January 2014) about checks on water quality and microbial load, including testing for *Legionella* spp. and the routine cleaning and disinfection of car wash systems [61]. In addition, the Australian Car Wash Association published a document for *Legionella* control at car wash systems soon after the first outbreak of legionellosis, recognizing a car wash in Hoppers Crossing, Victoria, as a source of infection [62] which caused the hospital admission of seven patients with a diagnosis of legionellosis. Furthermore, other two documents, a Public Health and Wellbeing Act and a Public Health and Wellbeing Regulation, were published in 2008 and 2009, respectively. These documents establish many different and general key points, including the storage of water at temperatures < 30 °C, the replacement of rubber hosing with polyethylene, metal or copper tubing and the regular disinfection of water systems with a chlorine-based disinfectant [63,64]. In Italy, the 2015 guidelines for the prevention and control of legionellosis [10] only includes the risk of the workers at car wash installations contracting legionellosis, and not their customers. Therefore, it does not provide any specific recommendations about measures that these structures can adopt to avoid the risk of *Legionella* contamination because only hospital water systems, air conditioning implants, cooling towers, spas, swimming pools and hot water systems are considered as common sources of infection.

At present, in the guide lines in force in Italy, there is no reference to the presence of *Legionella* in aerosols, our belief is that, in future revisions of the guidelines, the monitoring of aerosol in risk environments must be inserted. It is clear that, as long as this is not implemented, the only way to limit the *Legionella* risk is to conduct a good assessment of the presence of germs in water and the careful application of diagnostic and innovative preventative actions to try to contain its diffusion as much as possible [65,66]. In addition, an improved characterization of the environmental exposures that facilitate the risk of *Legionella* transmission may provide a rational for focusing prevention efforts, and may also help us to better recognize the source of infection. Indeed, making a good map of *Legionella* infections detected from a specific environmental source could play a leading role in the epidemiological investigation.

## 5. Conclusions

*Legionella* spp. is now considered an important waterborne pathogen able to contaminate any natural and artificial water system. Naturally, the hospital setting is the most studied setting and it has been evaluated in all its aspects especially due to the presence of immunocompromised patients (elderly people, patients affected by malignancies and other chronic diseases). However, now legionellosis is actually also considered an occupational risk and different work environments have been studied before now. Car washes are an unusual location, rarely considered in studies, but that can certainly have an importance in spreading the disease considering that, during the work activity, a lot of aerosols are formed that can be inhaled by both workers and customers, resulting in a real risk of contracting the infection. The knowledge about this setting is very limited due to the very poor presence of researchers in the international literature. We think that our study has the merit to widen and improve this knowledge and it represents another step forward in the fight against legionellosis.

## 6. Limits of the Study

As described in the literature, the exposition of environmental conditions, such as the chlorination of municipal water supplies, favors the formation of resistant and potentially infectious viable but non-culturable (VBNC) *Legionella* that cannot be detected using the standard culture. For this reason, the data relating to the isolated bacterial load could be underestimated. Likewise, in the case of very low charges, there may be false negative results.

## Figures and Tables

**Table 1:** Distribution of water and aerosol samples collected from car wash facilities vs. sampling day; positivity (%) and serogroups identified. CFUs/L and CFUs/m^3^ min/max were also reported.

**Sampling** **Days**	**Total Number** **of Samples (n)**	**Positive Samples (n; % POS)**	***Legionella*** **Serogroups (n)**	**CFUs/L** **(Min–Max)**
**Water**				
**Monday**	30	6 (20)	*L. pneumophila* 1 (1) *L. pneumophila* 10 (3) *L. gormanii (2)*	100 200–1000 200–800
**Thursday**	30	1 (3.3)	*L. pneumophila* 7 (1)	500
**Total**	60	7 (11.7)		
**Sampling** **Days**	**Total Number** **of Samples (n)**	**Positive Samples (n; % POS)**	***Legionella*** **Serogroups (n)**	**CFUs/m^3^** **(Min–Max)**
**Aerosol**				
**Monday**	30	3 (10)	*L. pneumophila* 10 (2) *L. gormanii* (1)	50–150 10
**Thursday**	30	0	---	---
**Total**	60	3 (5)		

## Data Availability

Data are contained within the article.
